# Investigation of chicken housekeeping genes using next-generation sequencing data

**DOI:** 10.3389/fgene.2022.827538

**Published:** 2022-09-13

**Authors:** Karim Hasanpur, Sevda Hosseinzadeh, Atiye Mirzaaghayi, Sadegh Alijani

**Affiliations:** Department of Animal Science, Faculty of Agriculture, University of Tabriz, Tabriz, Iran

**Keywords:** housekeeping genes, chicken, New Tuxedo, RNA-sequencing, coefficient of variation

## Abstract

Accurate normalization of the gene expression assays, using housekeeping genes (HKGs), is critically necessary. To do so, selection of a proper set of HKGs for a specific experiment is of great importance. Despite many studies, there is no consensus about the suitable set of HKGs for implementing in the quantitative real-time PCR analyses of chicken tissues. A limited number of HKGs have been widely used. However, wide utilization of a little number of HKGs for all tissues is challenging. The emergence of high-throughput gene expression RNA-seq data has enabled the simultaneous comparison of the stability of multiple HKGs. Therefore, employing the average coefficient of variations of at least three datasets per tissue, we sorted all reliably expressed genes (REGs; with FPKM ≥ 1 in at least one sample) and introduced the top 10 most suitable and stable reference genes for each of the 16 chicken tissues. We evaluated the consistency of the results of five tissues using the same methodology on other datasets. Furthermore, we assessed 96 previously widely used HKGs (WU-HKGs) in order to challenge the accuracy of the previous studies. The New Tuxedo software suite was used for the main analyses. The results revealed novel, different sets of reference genes for each of the tissues with 17 common genes among the top 10 genes lists of 16 tissues. The results did disprove the suitability of WU-HKGs such as *Actb*, *Ldha*, *Scd*, *B2m*, and *Hprt1* for any of the tissues examined. On the contrary, a total of 6, 13, 14, 23, and 32 validated housekeeping genes (V-HKGs) were discovered as the most stable and suitable reference genes for muscle, spleen, liver, heart, and kidney tissues, respectively. Although we identified a few new HKGs usable for multiple tissues, the selection of suitable HKGs is required to be tissue specific. The newly introduced reference genes from the present study, despite lacking experimental validation, will be able to contribute to the more accurate normalization for future expression analysis of chicken genes.

## Introduction

Housekeeping genes (HKGs), by definition, are genes required for the maintenance of basal cellular function, irrespective of their specific roles in the tissue or organism. HKGs are expected to express stably in all tissues of an organism under different conditions, regardless of developmental stage, sex, or external stressors (e.g., heat stress, disease, and immunological challenge, among others). Full characterization of a minimal set of genes that are required to sustain the life of a tissue is of particular interest (Eisenberg and Levanon, 2013). The current trend of analyses of global gene expression data using microarray or RNA-seq technologies has enabled the simultaneous analysis of tens of thousands of genes. However, quantitative real-time PCR (qPCR) has remained the only valid, more preferred independent tool for validating the results of genome-wide gene expression analyses (VanGuilder et al., 2008). The reliability of the final quantification result of qPCR depends heavily on the utilization of one or multiple internal reference genes for the normalization of the expression of the genes of interest. Normalization to a set of HKGs is nowadays a current and crucial procedure and is preferred to the normalization to a single reference gene. Therefore, the identification of at least two proper, stable HKGs for a specified tissue is crucial (Bagés et al., 2015).

The simultaneous analysis of a large number of genes was not possible until the emergence of high-throughput next-generation sequencing data. Although the evaluation of expression stability of potential reference genes has been carried out earlier for several tissues of chicken (Yang et al., 2013; Olias et al., 2014; Oliveira et al., 2017; Hassanpour et al., 2018; Zhang et al., 2018), the methods of choice of almost all of them were merely based on the qPCR and utilization of the BestKeeper (Pfaffl et al., 2004), geNorm (Vandesompele et al., 2002), and NormFinder (Andersen et al., 2004) statistical algorithms. To our knowledge, the work on identifying suitable HKGs using high-throughput microarray or RNA-seq data is scarce and limited to only some plant species including olive and *Arabidopsis* (Zhuo et al., 2016; Carmona et al., 2017), grapevine (González-agüero et al., 2013), white campion (Zemp et al., 2014), and some insects and animals including sweet potato whitefly (Su et al., 2013), Arctic charr (Pashay Ahi et al., 2013), human (Carmona et al., 2017), and human and mouse (de Jonge et al., 2007). However, there is no comprehensive study to address the most suited HKGs in chicken using RNA-seq data. In the present work, we tested most of the reliably expressed genes (REGs) for stability in the 16 important chicken tissues using at least three RNA-seq datasets per tissue and reported 10 most stably expressed genes for each of them to be used as proper sets of HKGs in the future gene expression assays. In addition, the consistency of the results was evaluated for five tissues, namely, heart, kidney, liver, muscle, and spleen. The evaluation step was not performed for the remaining 11 tissues as a sufficient, required number of high-depth datasets were not available.

## Materials and methods

### Selection of the desired tissues for study

Based on the importance of the tissues in research and availability of sufficient gene expression data, we selected 16 chicken tissues, namely, adipose, blood, brain, bursa of Fabricius, duodenum, heart, ileum, jejunum, kidney, liver, lung, muscle, ovary, skin, spleen, and trachea, for the current work. There were no sufficient datasets (at least three) for other tissues in the databases.

### RNA-seq data collection from databases

The required RNA-seq datasets were downloaded from NCBI (https://www.ncbi.nlm.nih.gov). At least three datasets were downloaded per tissue. Each dataset should have at least three replicated samples for case group and at least three replicated samples for control or another treatment group. The treatment of the case samples was not important as the only criterion for the comparison was to compare the expression of genes between case (treated) and control (untreated) or another treated group. For simplicity, the two mentioned groups will be called case and control, hereafter. The data were downloaded in SRA format using the SRA Toolkit and converted into fastq format using the fastq-dump tool. In Table 1, the accession numbers are shown, and in Supplementary Table S1, the meta information of the used datasets are presented.

**TABLE 1:** Accession numbers of the used datasets for discovering the most stably expressed genes for 16 chicken tissues.

Training datasets	Adipose	SRP143406	SRP343295	SRP042257	SRP212250		
	Blood	SRP200185	SRP200118	SRP200118	SRP310357	SRP310357	
	Brain	SRP102082	SRP081121	SRP233052	SRP233052		
	Burs	ERP122030	ERP122030	SRP163233	SRP098825		
	Duodenum	SRP348148	SRP299602	SRP173587	SRP055561		
	Heart	SRP265642	SRP097223	SRP153755			
	Ileum	SRP149780	SRP200118	SRP300399	SRP126304		
	Jejunum	SRP280208	SRP140601	ERP121879	ERP121879		
	Kidney	SRP097223	SRP092600	SRP338989			
	Liver	SRP143406	SRP097223	SRP321387	SRP294224	SRP161836	SRP133195
	Lung	SRP097223	SRP265640	SRP233531	SRP238721	SRP081121	
	Muscle	SRP217060	SRP217060	SRP217060	SRP217060	SRP159467	SRP321387
	Ovary	SRR12315154	SRP143406	SRP256253	SRP256253		
	Skin	SRP343295	SRP142597	SRP126033	SRP112878		
	Spleen	SRP097223	SRP225741	SRP174144	SRP280208	SRP158365	SRP174144
	Trachea	SRP338989	SRP247563	SRP226600	SRP126851		
Evaluation datasets	Heart	SRP152925	SRP266037	SRP159467			
	Kidney	SRP338989	SRP338989	SRP338989			
	Liver	SRP111815	SRP104528	SRP233052	SRP233052	SRP081121	SRP100368
	Muscle	SRP255211	SRP104528	SRP327337	SRP327185	SRP313854	SRP226900
	Spleen	SRP254842	SRP254842	SRP223412	SRP173965	SRP174144	SRP174144

### Required conditions for the datasets to be chosen for the analyses

The main question of the current work was “Which HKGs are the most suitable?” It is obvious that genes with the lowest expression differences between the case and control groups within a specified dataset as well as with expression sustainability among all datasets of a specified tissue could be considered as the most suitable HKGs. Since at least three datasets were analyzed per tissue, genes with the highest consistency of expression across all the experiments were finally introduced as the most proper sets of HKGs. We screened the NCBI database exhaustively to download only the datasets that address the question of research. Therefore, we downloaded only the datasets with the following conditions: 1) Illumina paired-end RNA-seq data (no single-end data used); 2) each of the tissues should have at least three datasets; 3) each dataset could be subset into only two case and control groups; and 4) each case group and control group should have at least three replicate samples.

### Analyses of individual datasets

**FIGURE 1:**
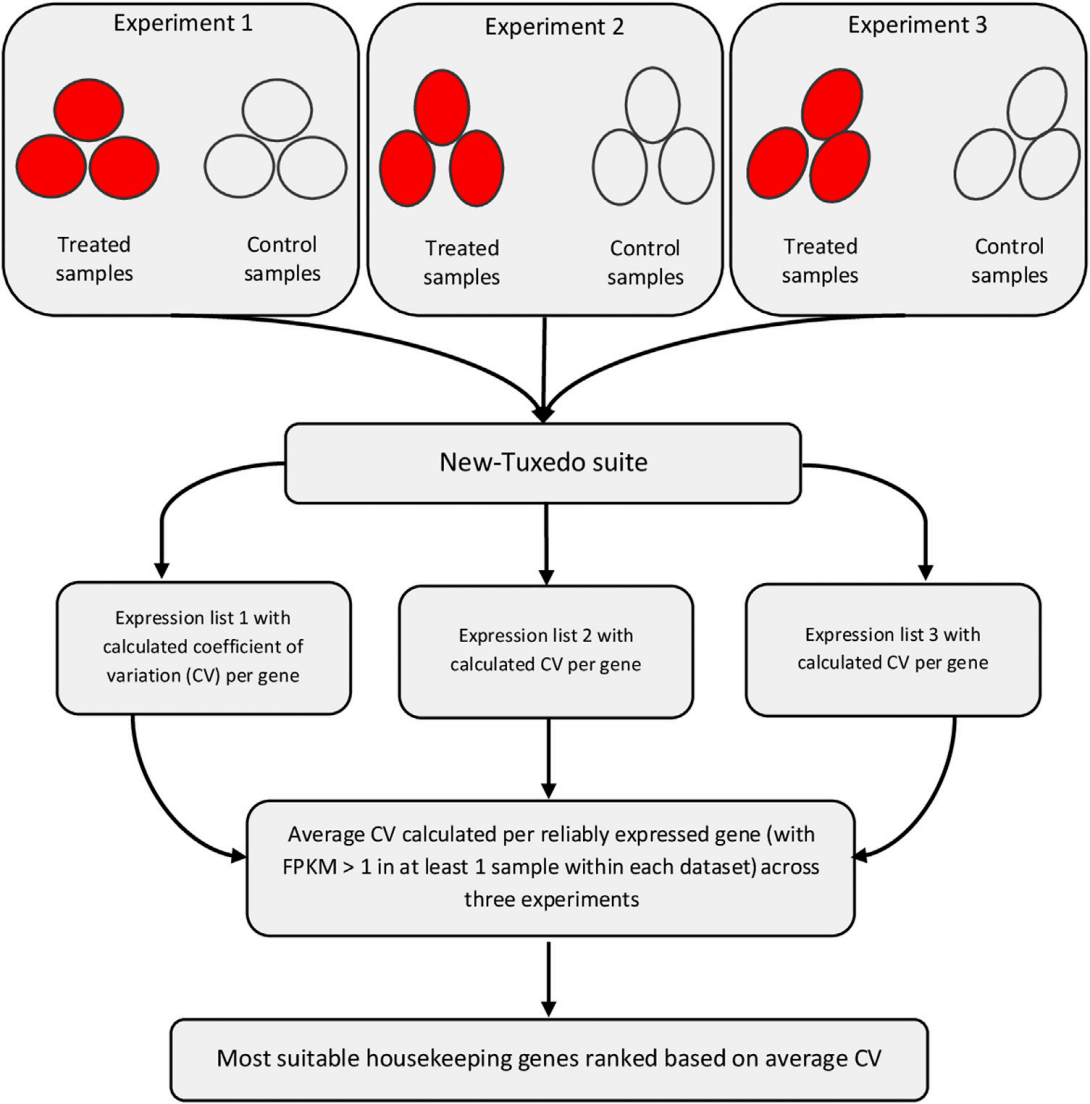
Flowchart of detection of the most suitable housekeeping genes for a specific tissue. This workflow was repeated for all tissues separately. At least three datasets were analyzed per tissue. The comparisons within each experiment were performed between the treated and control groups, each with at least three replicates. All the used datasets are publicly available data generated using Illumina paired-end RNA-Sequencing method. Software programs within the New Tuxedo suite were hisat2 (for mapping of reads onto reference genome), Stringtie (for assembly and read counting), and Cuffdiff (for differentiall expression analysis). Expression stability was monitored per gene based on the coefficient of variation (CV), and ranking of most stably expressed genes were performed based on the average CV criterion.

The individual datasets were analyzed separately using the New Tuxedo software suite employing the Ensembl Gallus gallus Build 6.0 reference genome (https://asia.ensembl.org/Gallus_gallus/info/index). At first, Fastqc (Andrews, 2010) and Trimmomatic (Bolger et al., 2014) software tools were employed for quality control and trimming, respectively. Datasets with insufficient quality metrics were excluded. The data were trimmed using ILLUMINACLIP, SLIDING WINDOW (window size 3–5 and Phred quality mean of 20–28), CROP (to trim 3–10 left-end nucleotides), AVGQUAL (minimum Phred quality of 20–25), and MINLEN (read length ≥ 40–45) options. Depending on the dataset, the values were varied. The Hisat2 software (Kim et al., 2019) (available at https://daehwankimlab.github.io/hisat2) was used for both indexing of the genome and mapping of the clean reads onto the indexed reference genome. The Stringtie software (Kovaka et al., 2019) (available at http://ccb.jhu.edu/software/stringtie) was used for assembly of transcripts of each sample using the -G option that forces the assembly to be limited to only the known genes. The transcripts of all samples of all experiments were assembled using the merge option of Stringtie. The Cuffdiff software (Trapnell et al., 2010) (available in http://cole-trapnell-lab.github.io/cufflinks) was used for differential expression analysis between the case and control groups with the multiread and bias correction options enabled. Genes with considerable expression differences between the two mentioned groups resulted in statistically significant differences, while genes with constant expressions between the two groups have *p*-values approximate to 1. Genes with unreliable expression (FPKM < 1) in all samples of a dataset were excluded. The expression (FPKM) values of all samples within a specified experiment were gathered, and mean, variation, and coefficient of variation (CV) statistics were calculated for each gene. Only REGs (i.e., genes passing the aforementioned filter in all datasets of a tissue) were considered for the discovery of the stably expressed genes. Then, average CV was calculated for each gene across all datasets within a specified tissue. Genes were sorted in ascending order and ranked based on the average CV, and those with the lowest average CV values were reported as the most stable and suitable housekeeping genes. The flowchart of the analyses is shown in Figure 1. This process was repeated for all 16 tissues, and the top 10 most stably expressed genes were reported for each tissue separately.

### Gene ontology and pathway enrichment analyses of the most stably expressed genes

All the top 10 genes of the 16 tissues were gathered, and the duplicated genes were deduplicated. In the end, a total of 139 unique genes were submitted to gene ontology and pathway enrichment analyses using the DAVID web-based software (Jiao et al., 2012) (available at https://david.ncifcrf.gov) in order to understand the functions and to gain insight into the pathways that the less variable genes are involved in.

### Evaluation of the consistency of the results

For five of the tissues, there were six or more datasets. For each of heart and kidney tissues, there were six datasets, and for each of liver, muscle, and spleen tissues, there were 12 datasets. We analyzed half of the datasets of the mentioned tissues as training and the second half for the evaluation of the consistency of the results of the training datasets. We named the second set as evaluation datasets. As for the training datasets, the REGs of the evaluation datasets were also ranked based on the average CV. The top 100 genes of the training datasets were compared with those of the evaluation datasets and those genes that were in common in both of the top 100 genes lists were reported as validated housekeeping genes (V-HKGs). The more the counts of matched genes between the two top 100 genes lists, the greater the accuracy and repeatability of discovering the stable genes.

### Assessment of the suitability of widely used housekeeping genes

A total of 96 WU-HKGs were selected from the literature. The list of WU-HKGs and their corresponding citations are shown in Supplementary Table S2. Gene expression analysis using a real-time PCR assay was the main subject of the reviewed papers. The main objective of the current section was to challenge the accuracy of the previously conducted gene expression studies that had used nonproper HKGs.

## Results and discussion

We used 94 datasets (70 for training and 24 for evaluation) sourced from 16 tissues, namely, adipose, blood, brain, bursa of Fabricius, duodenum, heart, ileum, jejunum, kidney, liver, lung, muscle, ovary, skin, spleen, and trachea. A total of 23,403 genes were analyzed in each dataset, and almost 3,000–11,000 REGs (with FPKM value ≥ 1 in at least one sample of a datasets) were assessed for the stability of expression. In contrast, nearly 50–85% of genes were filtered out because of the inconsistency of expression or because of low coverage of sequencing in some of the datasets. It is worth mentioning that only REGs of all datasets within a tissue were allowed for the final analysis. For tissues with more available datasets (such as spleen, liver, and muscle), we only chose the datasets with sufficiently deep sequencing, because the low coverage datasets would not guarantee the possibility of the evaluation of all REGs. In contrast, for less studied tissues that had no abundant datasets available, we decided to utilize all available datasets regardless of their sequencing depths. It is obvious that the insufficient coverage of datasets will cause the number of analyzed genes to be reduced. The total number of datasets and REGs for each of the tissues are reported in Supplementary Table S3.

**FIGURE 2:**
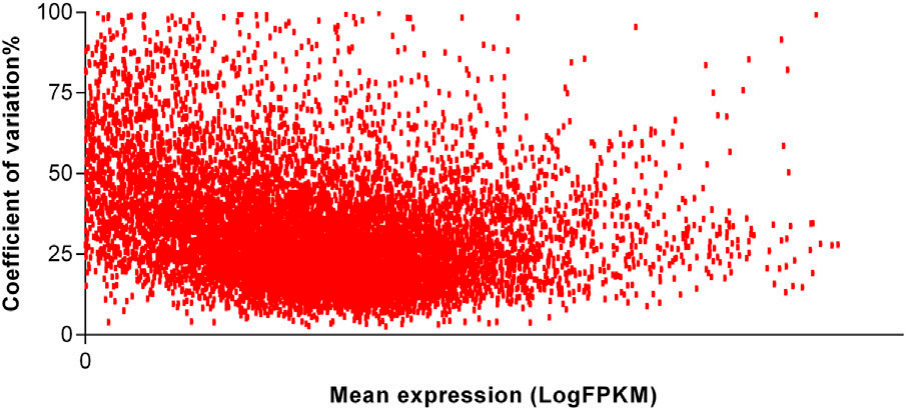
Scatterplot of the reliably expressed genes of one dataset of adipose tissue. The relationship of mean (in log10 scale) and variation of expression of genes were assessed in order to understand whether the variability of expression is increased with the mean of expression. The expression values are given as fragments per kilobase of transcript per million reads in log10 scale.

To identify the most stable reference genes, we first checked the relation of mean of expression and variation. We observed no relation between the mentioned coefficients, and thereby, the suggested reference genes can be used for the normalization of the interested genes irrespective of their expression levels. In Figure 2, a scatterplot demonstrating the relation of mean and CV of one experiment of adipose tissue is shown. Scatterplots of the remaining experiments were similar and therefore were not shown here.

The methodology used in the present work to find the most stable reference genes revealed interesting results. Almost all of the introduced reference genes (i.e., top 10 most stably expressed genes) were new, indicating that the previously used HKGs were not as stable as required for a gene to be considered as a proper HKG. Only eight (8.3%) of the WU-HKGs were present in the top 10 lists of five tissues including *Rpl4* for adipose; *Oaz1*, *Rpl27a*, and *Gapdh* for blood; *Rpl6* for jejunum; *Gusb* and *Polr2b* for kidney; and *Ap2m1* for liver. The top 10 most stably expressed genes introduced here for the remaining tissues were completely novel. In Table 2, the top 10 most stable reference genes are reported for the studied tissues.

**TABLE 2:** Top 10 most suitable reference genes for chicken tissues based on average coefficient of variations (CVs) across at least three experiments.

Adipose	Blood	Brain	Bursa	Duodenum	Heart	Ileum	Jejunum	Kidney	Liver	Lung	Muscle	Ovary	Skin	Spleen	Trachea
Abcb6	Psma1	Serbp1	Rap2c	Amot	Mrpl33	Dhx30	**Atp5b**	Itgb1bp3	Fam120a	Atl1	Adam17	**Tasor2**	**Hnrnpab**	Wdr81	**Tasor2**
Prrc2c	*Gapdh*	Nr1h3	Tmem259	**Xpo5**	**Ilf2**	Dhx38	*Rpl6*	Uck1	Xpo6	Tfip11	Slc39a3	Pcif1	Ddb1	Hdac1	**Atl1**
Ubr7^a^	*Oaz1*	Bet1l	Cnot9	**Exosc10**	Rufy3	Arnt	**Gnb2l1**	*Polr2b*	**Ubr7**	Mvb12a	**Cops7a**	Tomm22	Lonp1	Scyl3	**Ikbkb**
Eif3a	Rpl39l	Arhgef9	Wasf2	Zyx	**Cep68**	Ckap2l	**Ilf2**	Col4a1	Mrps25	Slc35a1	Xpo7	Erlin1	Tcf25	Zc3h11b	**Cep68**
Pabpc1	Tmed10	**Atp5b**	**Grb2**	Usp5	Nfyc	**Hnrnpab**	Lasp1	*Gusb*	*Ap2m1*	Pisd	Fem1b	Poll	**Grb2**	Dnajc5	Cfap92
Tmem57	*Rpl27a*	Nono	Ascc2	Cnot1	Cuedc2	Nup188	Mif4gd	**Atl1**	Pcbd1	Phc1	Ipo9	**Ctnna1**	Mtmr3	Nek9	Parp9
Thrap3	Cox7b	Sumo3	Rpl7l1	Psmd13	**Tasor2**	Stx10	**Ikbkb**	Hbp1	Rnf130	Spout1	**Gsr**	Kbtbd4	Baz1b	Gzf1	Hspd1
*Rpl4*	Rpl7a	Rps3a	Man2c1	Fhl3	Slc7a3	**Eif2b5**	**Atl1**	Herc2	Adat1	**Tor1b**	Copg2	Brd8	**Exosc10**	Cdk12	Cd80
**Ctnna1**	Rpsap58	Uso1	Tpm3	Med1	**Gnb2l1**	Map3k14	Mkrn2	Ndufb9	Myh9	Cog5	Arcn1	Gsr	**Cops7a**	**Xpo5**	Mettl21c
Kcnh4	Eif4ebp1	Cd99l2	Chmp1a	**Eif2b5**	**Ak2**	Nol9	Aamp	**Tor1b**	Arpc2	**Cep68**	Eya3	Clpx	Yipf3	Mpp1	Mrpl40

a Highlighted (bold) genes are in common for at least two tissues. Underlined genes are among the widely used housekeeping genes.

As can be seen in Table 2, some of the introduced reference genes (17 genes) were in common in the top 10 lists of at least two tissues. For example, *Atl1* was identified as suitable for four tissues. *Cep68* and *Tasor2* genes were in common in the lists of three tissues, and *Ubr7*, *Ctnna1*, *Atp5b*, *Grb2*, *Xpo5*, *Exosc10*, *Eif2b5*, *Ilf2*, *Gnb2l1*, *Hnrnpab*, *Ikbkb*, *Tor1b*, *cops7a*, and *Gsr* genes were in common in the top 10 lists of two tissues. It is obvious that genes with more frequent occurrence in multiple top 10 genes lists are more likely suggested as suitable HKGs than are those with only one occurrence. Therefore, these 17 genes are strongly suggested to be used as HKGs for the mentioned tissues.

### Gene ontology and Kyoto Encyclopedia of Genes and Genomes pathway analyses of the most stably expressed genes

Understanding the functionality of the most stable reference genes is of great importance. In the current work, the top 10 most stable genes of all 16 studied tissues (totally 134 unique genes) were subjected to gene ontology and KEGG pathway enrichment analyses using the DAVID web tool. The results revealed that the most stably expressed genes that enriched significantly (*p* < 0.05) in molecular function terms, biological processes terms, and KEGG pathways were generally related with the transcription and translation of proteins (e.g., *RNA* binding, protein binding, structural constituent of ribosome, translational initiation, positive regulation of transcription, cytoplasmic translation, nucleocytoplasmic transport, RNA degradation, and ribosome, among others). In other words, as compared to genes of other processes or pathways, it seems that genes associated with transcription and translation are less variable under different conditions and are more suited as HKGs. These results are in accordance with the findings of Kouadjo et al. (2007) and de Jonge et al. (2007), who reported the protein biosynthesis–related genes as the most stably expressed genes. Gene ontology terms as well as KEGG pathways enriched by the top 10 most stably expressed genes are reported in Supplementary Table S4.

### Consistency of the results of the training datasets

As mentioned above, other sets of data of heart (three datasets), kidney (three datasets), liver (six datasets), muscle (six datasets), and spleen (six datasets) tissues were analyzed in the same way as the training datasets in order to evaluate the consistency of the results of the training datasets. The average CVs of the training datasets strongly correlated with that of the evaluation datasets with Pearson correlation coefficient ranging from 0.64 to 0.88 and Spearman correlation coefficients ranging from 0.67 to 0.82, indicating the relatively high accuracy of the discovering stable genes. The top 100 genes of the training datasets were compared with that of the evaluation datasets. We found that the top 100 genes of the training datasets also took place in higher ranks in the evaluation datasets. Except for muscle, we found relatively consistent results for the remaining four tissues. There were 6, 13, 14, 23, and 32 genes in common in the top 100 genes of the training and evaluation datasets of muscle, spleen, liver, heart, and kidney tissues, respectively. In total, 80% of the top 100 genes for kidney tissue and almost 50% of the top 100 genes for heart, liver, and spleen tissues were present in the top 500 genes of the evaluation datasets. It should be noted that in the case of random distribution of genes in the various ranks of the evaluation datasets, less than 10% of the top 100 genes of training datasets would be present in the top 500 genes of the evaluation datasets [(500/number of analyzed genes) × 100 = ∼4.6–8%]. These findings indicate that the identification of the most stably expressed genes with the used method is, to some extent, accurate, and the repeatability of the results is considerable. In Table 3, the number of common genes in the top 100 genes of the training datasets and top 500 genes of the evaluation datasets are presented. Although the employed approach identified reliable, stable reference genes for all tissues, we recommend the utilization of the reported HKGs cautiously. In addition, we invite related researchers to further validate the reported HKGs using real-time PCR as this was not possible in the current work.

**TABLE 3:** Number of genes that are in common in the top 100 genes list of the training datasets and the top 100 to top 500 genes lists of the evaluation datasets.

	Heart	Kidney	Liver	Muscle	Spleen
Top-100 genes of evaluation datasets	23	32	14	6	13
Top-200 genes of evaluation datasets	38	51	26	11	21
Top-300 genes of evaluation datasets	48	68	36	16	31
Top-400 genes of evaluation datasets	59	76	46	19	35
Top-500 genes of evaluation datasets	63	80	49	23	43

**FIGURE 3:**
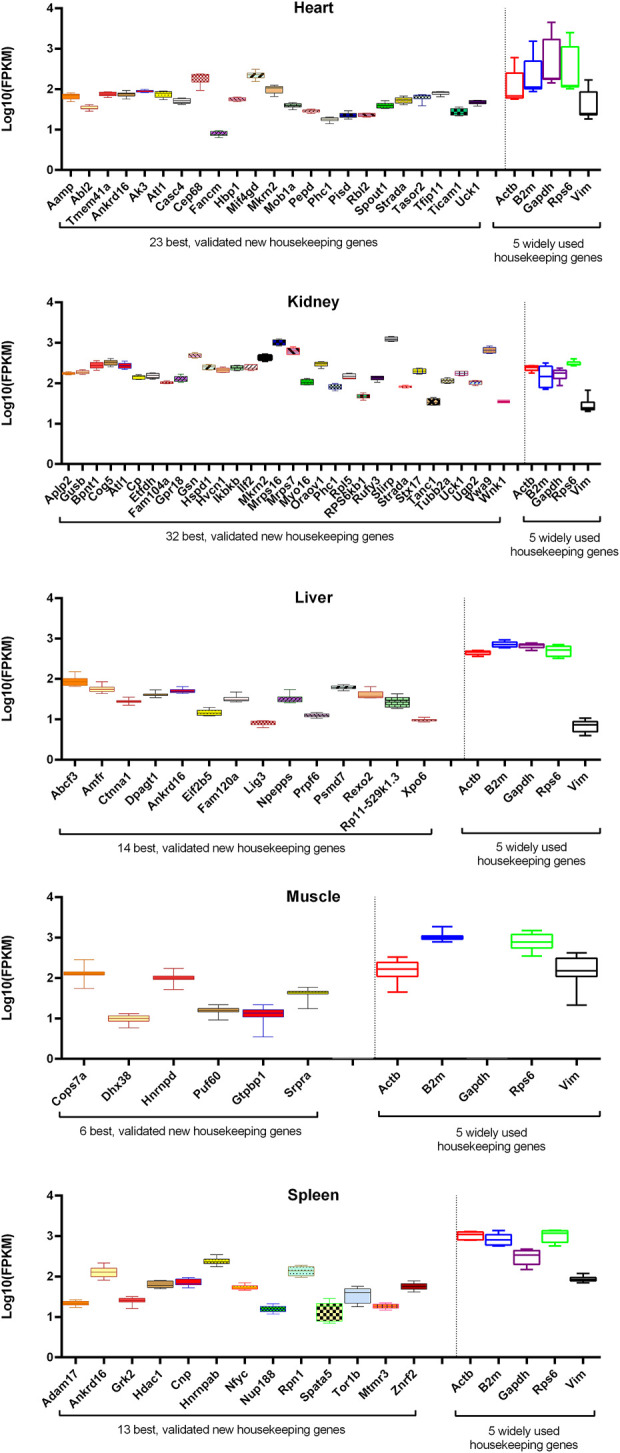
Box plots of the expression of most stable, validated housekeeping genes in comparison with that of five randomly selected widely used housekeeping genes of five chicken tissues. For each tissue, the expression variation of newly introduced housekeeping genes (left side of the vertical line) was compared with that of five old and widely used housekeeping genes (right side of the vertical line). The expression values are given as fragments per kilobase of transcript per million reads in log10 scale (whiskers: min to max).

Here, we introduced only those genes that were in common between the top 100 genes of the training and evaluation datasets as V-HKGs. Six of the V-HKGs, namely, *Ankrd16*, *Strada*, *Phc1*, *Atl1*, *Mkrn2*, and *Uck1*, were observed commonly for at least two tissues. *Ankrd16* gene was identified as suitable for heart, liver, and spleen tissues. The remaining five genes were identified as the best for both heart and kidney tissues. In Table 4, the official names of the V-HKGs are reported. In addition, in Figure 3, the boxplots of the V-HKGs are illustrated. As can be seen in Figure 3, the expression variations of the V-HKGs were negligible.

**TABLE 4:** Most stably expressed genes of five chicken tissues that are in common between the top 100 genes of the training dataset and the top 100 genes of the evaluation dataset.

Heart	Kidney	Liver	Muscle	Spleen
Mob1a	Ikbkb	Rp11-529k1.3	Srpra	Nfyc
**Strada**	Wnk1	Dpagt1	Cops7a	Hdac1
Ticam1	Aplp2	Rbm7	Hnrnpd	Cnp
Cep68	Ilf2	Npepps	Dhx38	Adam17
**Phc1**	**Strada**	Eif2b5	Gtpbp1	Spata5
**Atl1**	Ugp2	*Ap2m1*	Puf60	Grk2
Fancm	Bpnt1	Amfr		Rpn1
Abi2	**Phc1**	Psmd7		Hnrnpab
Aamp	Etfdh	Fam120a		Mtmr3
Tmem41a	Rufy3	Ctnna1		Nup188
Ak3	Oraov1	Xpo6		Tor1b
Hbp1	Slirp	Lig3		Znrf2
Casc4	**Atl1**	**Ankrd16**		**Ankrd16**
Rbl2	Hspd1	Prpf6		
Pepd	Tanc1			
**Mkrn2**	*Rpl5*			
Tfip11	Cp			
Pisd	Gpr18			
Spout1	Cog5			
**Uck1**	Vwa9			
Mif4gd	**Mkrn2**			
**Ankrd16**	Hvcn1			
Tasor2	**Uck1**			
	Gsn			
	Fam104a			
	Mrps7			
	*Gusb*			
	Rps6kb1			
	Myo19			
	Tubb2a			
	Stx17			
	Mrps16			

a Highlighted (bold) genes are in common for at least two tissues. Underlined genes are among the widely used housekeeping genes.

### Assessing the suitability of 96 widely used housekeeping genes

In Supplementary Tables S5 and S6, the average CVs and rankings of the WU-HKGs among the REGs are reported, respectively. The expression profile of most of the 96 WU-HKGs showed inconsistency in some of the chicken tissues. Eight genes (i.e., *Mb*, *Dimt1*, *Rps29*, *Stx5*, *Gys1*, *Il6*, *Rbx1*, and *Rnasek*) showed no expression in more than five tissues. It is obvious that the mentioned genes do not have merit to be suggested as proper HKGs, although some of them have been traditionally used widely for the normalization of real-time PCR assays. In contrast, only 13 genes showed consistent expression in all 16 tissues, including *Ap2m1*, *Gusb*, *Polr2b*, *Rpl6*, *Eif4a3*, *Rpl4*, *Eef1a1*, *Gapdh*, *Rpl19*, *Rpl27a*, *Rpl31*, and *Rps6*. Six out of the 13 mentioned genes (i.e., *Eef1a1*, *Gapdh, Rpl19, Rpl27a*, *Rpl31*, and *Rps6*) showed relatively consistent, stable expression and ranked among the best 100 most stably expressed genes of two tissues. Although not detected in three datasets, *Ap2m1* performed well in seven tissues, which was followed by *Gusb*, *Polr2b*, *Rpl6*, *Rpl*4, and *Eif4a3*. These WU-HKGs were high-ranked in three or more tissues and seem to be suitable for further utilization.

For blood, 17 WU-HKGs were relatively suitable, which was followed by adipose (seven genes), jejunum (six genes), kidney (six genes), and liver (five genes), indicating that the WU-HKGs are more suitable for blood than for other tissues. For the remaining tissues, almost none of the WU-HKGs is suitable, and further use of them is not suggested.

The variation of expression of the 96 WU-HKGs were considerably more than that of the stable reference genes that were introduced in the present work. In Figure 3, the expression variations of five randomly selected WU-HKGs (i.e., *Gapdh*, *Actb*, *Vim*, *Rps6*, and *B2m*) are illustrated. As can be seen in Figure 3, some of the mentioned genes were not stably expressed across all experiments of 16 tissues. Two of them (i.e., *Gapdh* and *Rps6*) were among the top 100 stable genes, while the remaining three (i.e., *Vim*, *B2m*, and *Actb*) were not.

Previous studies have reported relatively high variability for the expression of WU-HKGs in a varied range of tissues and organs in livestock animals (Gromboni et al., 2020; Lozano-Villegas et al., 2021) as well as in humans (Mahoney et al., 2004; Caracausi et al., 2017; Cadenas et al., 2022), mice (Fu et al., 2020; Muñoz et al., 2021), and insects (Shi et al., 2016), among others.

For adipose tissue, only *Rpl4* gene took place within the top 10 stable genes among 10,343 REGs (Table 2). Other WU-HKGs were not even within the best 100 genes. We found it interesting that having fold changes of ∼9.7 and 12.0, respectively, *Acta1* and *Scd* genes showed significant difference between the case and control groups in one experiment of adipose (q-value < 0.05). Therefore, these genes are no longer suggested for further utilization. Unlike *Rpl4* gene, which is also proposed here, *Tbp* has previously been reported elsewhere as a suitable reference gene for the normalization of gene expression data of adipose tissue (Wang et al., 2020). *Tbp* was the only WU-HKG that was reliably expressed in all 16 tissues with nonsignificant differential expression in all of the 94 studied datasets. However, *Tbp* ranked 1,092 among the 10,343 REGs of adipose tissue. Other studies have reported *Rpl32* and *B2m* genes as two suitable HKGs for abdominal fat, compared with the other three genes, namely, *Sdha*, *Tbp*, and *Ywhaz* (Bagés et al., 2015). Neither of them, however, were identified as suitable HKGs for adipose in the current work. Na et al. (2021) compared 14 chicken reference genes and reported both *Tbp* and *Hmbs* genes as the most stably expressed genes during the growth and development of abdominal adipose tissue of broilers. They also reported *Tbp* and *Rpl13* genes as the most stable during the differentiation of primary preadipocytes and *Tbp* and *Hmbs* genes in preadipocytes and mature adipocytes.

In the present work, as compared to those of other tissues, greater number of WU-HKGs were relatively constant across all datasets of blood. Studying human peripheral blood, Martínez-Sánchez et al. (2019) found *Hprt* and *Tbp* as the most reliable genes. They suggested the utilization of *Gapdh*, *B2m*, and *Rpl13a* genes to be avoided. Likewise, Dheda et al. (2004) emphasized the avoidance of employment of *Gapdh*, *B2m*, and *Actb* genes (each with ∼10- to 30-fold variability across conditions) for normalizing mRNA levels in human pulmonary tuberculosis. For blood, we found the following four WU-HKGs as relatively stable: *Gapdh* (rank = 2), *Rpl13* (rank = 80), *Oaz1* (rank = 3), and *Rpl27a* (rank = 6).

For heart tissue, except *Gusb* (rank = 50) and *Tbp* (rank = 57) genes, further utilization of other WU-HKGs genes is not suggested. *TBP* has been also identified as a suitable reference gene for lung and heart (Hassanpour et al., 2018) and abdominal fat (Wang et al., 2020) tissues. Because of the low coverage of the used datasets of heart tissue in the current work, only 3,334 (6,235) genes in the training (evaluation) datasets were reliably expressed. Therefore, as expected, many of the WU-HKGs with little expression were not detected in heart datasets. On that account, only 33 WU-HKGs were analyzed. Gromboni et al. (2020) assessed the suitability of eight HKGs for heart. Their studied WU-HKGs were present in neither the top 10 genes nor the list of 23 V-HKGs of heart tissue in the current study. Hassanpour et al. (2018) investigated a panel of nine HKGs and introduced *Ywhaz* and *Rpl13* genes as suitable for chicken heart (Hassanpour et al., 2018). All of their studied genes were filtered out in the current study as lacking the criteria we employed to categorize the stable reference genes.

For kidney tissue, only *Polr2b* and *Gusb* genes (rankings of 3 and 5, respectively) and *Eef1a1*, *Nelfcd*, *Rpl5*, and *Rpl6* (rankings of less than 100) outperformed other WU-HKGs within 7,527 REGs. On the contrary, *Il6*, *Scd*, and *Dimt1* were among the worst genes and identified as the most inappropriate HKGs for kidney. Other WU-HKGs were also not stable and therefore not recommended for further use.

For liver tissue, only *Ap2m1* gene appeared suitable with a ranking of 5 among 8,428 REGs. Out of 94 analyzed datasets, *Ap2m1* was reliably expressed in 80, while it was significant (q-value < 0.05) in only two datasets belonging to blood and brain tissues. Therefore, although not suggested for brain, blood, heart, and lung tissues, *Ap2m1* seems to be suggested for more than one tissue. In yellow feathered broilers, Zhang et al. (2018) reported *Rpl13* gene as the most proper HKG for liver, compared with only six other candidate genes. In another research, *Ywhaz* and *Tbp* were found more stable than *B2m*, *Rpl32*, and *Sdha* genes (Bagés et al., 2015). In the present work, being significant in one, two, two, and three experiments among 12 experiments of liver, *Actb*, *B2m*, *Gapdh*, and *Rpl13* genes, respectively, were not proved to be suitable HKGs.

For lung tissue, only 34 WU-HKGs showed reliable expression. Almost 95% of these 34 genes were not stable. Only *Gusb* (rank 30 among 3,562 REGs) was identified as relatively suitable. In an attempt, Kriegova et al. (2008) investigated 10 candidate HKGs and introduced *Rpl32* as the most suitable HKG for lung tissue. Being excluded from the analyses in the filtration steps, *Rpl32* was not identified as a suitable HKG in the current work for neither lung tissue nor other tissues. The results of Fu et al. (2020) indicated that none of the 15 WU-HKGs that they studied were sufficiently good as reference genes. However, they suggested the combination of *Grcc10* and *Ppia* genes as a proper choice for the lung tissue of mouse infected with IAV.

The datasets of muscle in the current work appeared very variable. Although we only used datasets belonging to pectoral major muscle tissue, as compared to other tissues, there were less genes in common between the training and evaluation datasets. It seems that sampling of the tissues of the different studies had been done differently and not from the same section of the pectoral major muscle. The six identified V-HKGs (i.e., *Srpra*, *Cops7a*, *Hnrnpd*, *Dhx38*, *Gtpbp1*, and *Puf60*), however, showed sufficiently less variability within the used 12 different datasets and seem to be a suitable set of reference genes for muscle. The findings of Nascimento et al. (2015) showed *Hmbs* and *Hprt1* genes as the most stable while *Tfrc* and *B2m* as the least stable reference genes for the pectoralis major muscle of chicken. Their results, also, revealed that *Hmbs* and *Hprt1* gene expression did not change owing to dietary variations and thus were recommended for accurate normalization of RT-qPCR data of chicken pectoralis major muscle. In our results, the best WU-HKG was MIF (rank = 89). *Hprt1*, although expressed nonsignificantly in all six training analyses of pectoral muscle and its fold change ranged 1.0–1.3 between the case and control groups, appeared relatively variable within the case or control groups of the evaluation datasets and ranked 1,172 among 10,745 REGs. In accordance with the findings of the current work, Barber et al. (2005) analyzed *Gapdh* expression in a panel of 72 human tissues and observed a 15-fold difference in *Gapdh* mRNA copy numbers between the skeletal muscle and the breast. Their results confirmed previous reports of the marked variability of *Gapdh* expression between tissue types. On the contrary, Mahoney et al. (2004) concluded that *B2M* and *ACTB* were the most stably expressed HKGs in human skeletal muscle following resistance exercise, while *B2m* and *Gapdh* were the most stable following endurance exercise.

For ovary, we discovered three genes (i.e., *Tasor2*, *Ctnna1*, and *Gsr*) within its top 10 genes that were also identified as the most stable for other tissues. These stable genes along with all the top 10 genes of brain, bursa of Fabricius, duodenum, ileum, jejunum, lung, skin, and trachea were completely new and, to our knowledge, are first reported in the present study. Hassanpour et al. (2019) reported *Ywhaz*, *Hprt1*, and *Hmbs* genes as most stable. They suggested the combination of *Ywhaz*, *Hprt1*, and *Hmbs* as the best set of reference genes for ovarian and uterine tissues of laying hens under control and heat stress conditions. Cadenas et al. (2022) found that the stability of all reference genes differs among ovarian cell types in humans. They identified *Actb* as the best reference gene for oocytes and cumulus cells and *B2m* for medulla tissue and isolated follicles. They concluded that using a single validated reference gene may be sufficient when the available testing material is limited. For the ovarian cortex, depending on culture conditions, *Gapdh* or *Actb* were found to be the most stably expressed genes. Their reported stable genes were not confirmed in the current work.

Similar to the tissues discussed above, we evaluated the 96 HKGs for spleen transcriptome data as well. Having a relatively low CV and high rank (58 among 9,904 genes), only *Rpl6* proved to be relatively suitable for spleen. In a previous study, 10 HKGs were assessed and 2 genes, i.e., *Tbp* and *Ywhaz*, were identified as the most suitable HKGs for spleen tissue (Khan et al., 2017). None of them, however, was in the list of best, high-ranked HKGs in the current work. For spleen, liver, and cecum of different-aged specific-pathogen-free layer chickens and commercial turkeys, Mitra et al. (2016) suggested *Rpl13* and *Tbp* as the most stable reference genes. They also observed a stable expression of *Rpl13* and *Tfrc* genes in the mentioned tissue samples of turkey. In the current work, both *Rpl13* and *Tfrc* genes were expressed differently between the case and control groups of two experiments of evaluation datasets (q-value < 0.05). Therefore, our results did not prove the consistency of expression of *Rpl13* and *Tfrc* genes. Likewise, we could not approve the suitability of spleen HKGs introduced by Khan et al. (2017) and Borowska et al. (2016).

To our knowledge, the present work is the first comprehensive study that investigated all REGs for 16 most important chicken tissues. Most of the previous studies have compared only a handful of HKGs in which the used genes were in common (Zinzow-Kramer et al., 2014; Bagés et al., 2015; Mitra et al., 2016; Khan et al., 2017; Hassanpour et al., 2018; Zhang et al., 2018; Gromboni et al., 2020). The employed methodologies of the mentioned studies were NormFinder, GeNorm, BestKeeper, RefFinder, and delta CT (Mitra et al., 2016). Each of them has its own strengths and weaknesses. In RefFinder, PCR efficiencies are not taken into account. The NormFinder software is influenced by sample size (Spiegelaere et al., 2015). GeNorm ranking for genes is based on the highest degree of similarity in their expression profile and does not take the amount of variation into account (Andersen et al., 2004). BestKeeper utilizes Pearson correlation analysis and is just valid for normally distributed data with a homogeneous variance. In general, the ranking of HKGs is different based on the output of RefFinder, NormFinder, GeNorm, and BestKeeper, and there is little overlap (Kou et al., 2017). Owing to the dynamic and high-throughput nature of the next-generation sequencing data, the methodology that was introduced in the current work seems to overcome the weakness of the previously used methods. Moreover, utilization of different datasets that belong to different studies, instead of real-time PCR data, is the superiority of the current work over the previous research. In addition, the integration of the results of at least three datasets per tissue seems to increase the reliability of the results.

## Conclusion

In the present work, we, for the first time, conducted a comprehensive genome-wide gene expression evaluation of 3,000–11,000 genes, analyzing 94 experiments in order to assess the suitability of previously known HKGs as well as to discover the most stable, new housekeeping genes for each of 16 chicken tissues. The results clearly revealed novel reference genes with more stable expressions in different experimental conditions. On the basis of the definition of ubiquitous and stable expression, our results suggest that no single gene qualifies as a real HKG. In addition, although we identified some genes that were suited for more than one tissue, most of the introduced new and validated HKGs were tissue specific. Thus, instead of one suitable HKG, we reported 10 high-ranked, stable genes for each tissue to provide future studies with more options to choose from. The identified new HKGs were predominantly involved in transcription, translation, and protein biosynthesis. There were 17 common HKGs that were suitable for more than one tissue. We strongly suggest them as well as the V-HKGs for normalization in all future qRT-PCR experiments. We believe that the results of the present work will contribute to more accurate normalization of chicken gene expression data, especially for the data of heart, liver, kidney, spleen, and muscle tissues, and that their results will be validated by analyzing additional sets of datasets.

## Data Availability

The original contributions presented in the study are included in the article/Supplementary Material, and further inquiries can be directed to the corresponding author.
