# Supplementary material for: Investigation of chicken housekeeping genes using next-generation sequencing data
Source: Front Genet. 2022 Sep 13;13:827538. doi: 10.3389/fgene.2022.827538 (PMC9514876; doi:10.3389/fgene.2022.827538)
Supplement: Supplementary file 2 [file Table1.DOCX]

| Supplementary Table 1. Meta information of the used datasets for the analyses | | | | |
| --- | --- | --- | --- | --- |
| Dataset accession number | Reference | Treatment | Samples information | Sequence length (PE) |
| **Adipose** |  |  |  |  |
| SRP143406 | (Wei et al., 2019) | effect of feeding regimes on energy metabolism and egg production | 3 broiler chickens under ad libitum vs 3 chickens under restricted feeding | 125-bp |
| SRP343295 | - | Comparison of chicken groups with high abdominal fat and chicken groups with low abdominal fat | 3 high abdominal fat groups vs 3 low abdominal fat groups | 150-bp |
| SRP042257 | (Roux et al., 2016) | identification of mRNA editing using mRNA and genomic DNA sequencing | 4 male vs 4 female | 101-bp |
| SRP212250 | (Jin et al., 2021) | exploring transcriptome of Gushi chicken abdominal adipose tissue at different stages | 3 chickens at 22 weeks vs. 3 chickens at 14 weeks | 150-bp |
| **Blood** |  |  |  |  |
| SRP200185 | - | Whole blood transcriptome analysis of laying hen fed with a dried olive pomace supplemented diet | 5 chicken fed with a dried olive pomace supplemented diet vs 4 control | 76-bp |
| SRP200118 | (Lee et al., 2021) | compare the effects of supplements with L-methionine and D-methionine on the expression of the transcriptome | 3 chickens fed L-methionine and 3 chickens fed D-methionine | 101-bp |
| SRP200118 | (Lee et al. 2021) | compare the effects of supplements with L-methionine and D-methionine on the expression of the transcriptome in broiler chickens under acute heat stress | 3 chickens fed L-methionine and 3 chickens fed D-methionine | 101-bp |
| SRP310357 | (Dai et al., 2021) | differences of avian CD4+CD8+ double-positive T cells and CD8+ T cells in ALV-J infected chickens | 3 CD8^low^ vs 3 CD8^high^ | 150-bp |
| SRP310357 | (Dai et al., 2021) | differences of avian CD4+CD8+ double-positive T cells and CD8+ T cells in ALV-J infected chickens | 3 CD8^medium^ vs 3 CD8^high^AA | 150-bp |
| **Brain** |  |  |  |  |
| SRP102082 | (Zhuo et al., 2017) | Identification of Frequent of the Specific allele Expression | 3 chicken Leghorn × Fayoumi cross vs 3 chicken Fayoumi × Leghorn cross | 75-bp |
| SRP081121 | (Li et al., 2019b) | Comparison of chickens under different altitudes | 6 chicken under 3300m and 6 chicken under 670m | 125-bp |
| SRP233052 | (Wang et al., 2019) | Exploring the evolution of cis- and trans-regulatory divergence in the chicken genome between two contrasting breeds | 6 Cornish vs 6 cross2 | 100-bp |
| SRP233052 | (Wang et al., 2019) | Exploring the evolution of cis- and trans-regulatory divergence in the chicken genome between two contrasting breeds | 6 White Leghorn vs 5 cross3 | 100-bp |
| **Bursa of fabricius** |  |  |  |  |
| ERP122030 | (Chanthavixay et al., 2020) | Transcriptome analysis in bursa tissue of chicken lines under NDV infection and heat stress | 4 control Fayoumi vs 4 Treated Fayoumi | 150-bp |
| ERP122030 | (Chanthavixay et al., 2020) | Transcriptome analysis in bursa tissue of chicken lines under NDV infection and heat stress | 4 control Leghorn vs 4 Treated Fayoumi | 150-bp |
| SRP163233 | (Zhang et al., 2018) | Exploring immune function in the chicken stress model | 3chicken under stress vs 3 control | 150-bp |
| SRP098825 | (Ou et al., 2017) | Exploring chickens infected with infectious bursal disease virus | 3 chicken with mock infection vs 3 chicken with infection with infectious bursal disease virus (IBDV) | 100-bp /125-bp |
| **Duodenum** |  |  |  |  |
| SRP348148 | (Zou et al., 2022) | Exploring effect Bacillus subtilis on inhibits intestinal inflammation and oxidative stress | 3 chicken infected Bacillus subtilis vs 3 control | 151-bp |
| SRP299602 | (Xiao et al., 2021) | Exploring pathways related to Feed efficiency | 3 chicken fed low residual feed intake (LRFI) vs 3 chicken fed high residual feed intake (HRFI) | 150-bp |
| SRP173587 | (Boo et al., 2020) | Exploring Immune Response of Chicken Intraepithelial lymphocyte natural killer cells to very virulent Infectious Bursal Disease Virus (vvIBDV) | 3 uninfected chickens vs 3 chickens infected by vvIBDV | 100-bp |
| SRP055561 | (Yi et al., 2015) | A better understanding of the underlying mechanisms implicated in residual feed intake | 3 chickens fed with low feed efficiency vs 3 chickens fed with high feed efficiency | 100-bp |
| **Heart** |  |  |  |  |
| SRP265642 | (Hou et al., 2021) | Transcriptomic expression profiles of SPF chicken-heart infected with fowl adenovirus | 3 uninfected chickens vs 3 chickens infected by Fowl adenovirus | 150-bp |
| SRP097223 | (Tang et al., 2017) | Comparison of adaptation chickens for high-altitude with not adaptation chickens for high-altitude | 3 chickens in high-altitude vs 3 chickens in low-altitude | 100-bp |
| SRP153755 | - | Exploring of differential gene expression in Kirin Domestic Chickens under heat stresses | 4 chickens under heat stress vs 4 control | 150-bp |
| SRP152925 | (Zhang et al., 2019) | Exploring of differential gene expression in Chickens under heat stresses | 4 chickens under heat stress vs 4 control | 150-bp |
| SRP266037 | (Yu et al., 2021) | Exploring genes involved in cadmium-triggered oxidative stress in chicken heart | 4 chickens involved in cadmium-triggered oxidative stress vs 4 control | 151-bp |
| SRP159467 | (Park et al., 2018) | Exploring the effect of Environmental stress on Ethiopia indigenous chicken breeds | 9 chickens not adapted to heat vs 9 chickens adapted to heat | 101-bp |
| **Ileum** |  |  |  |  |
| SRP149780 | (Geng et al., 2020) | Exploring the role of carnosic acid on the expression of a panel of genes involved in lipid metabolism and bile acid metabolism | 4 IC vs 4 IT | 35-bp /150-bp |
| SRP200118 | (Lee et al., 2021) | Exploring the effect of heat stress if fed supplementation with L-methionine and D-methionine on the expression of the transcriptome | 6 chickens under heat stress vs 6 control | 101-bp |
| SRP300399 | (Chen et al., 2021) | Exploring evaluate the effects of dietary supplemental magnolol and honokiol in broilers infected with S. pullorum | 6 chickens infected by S. pullorum vs 6 control | 150-bp |
| SRP126304 | - | compare the transcriptome difference of broiler chickens with different ileum-thickness | 8 TK vs 5 TN | 150-bp |
| **Jejunum** |  |  |  |  |
| SRP280208 | - | Comparison of highest and lowest dropping moisture in chickens | 4 chickens under highest dropping moisture vs 3 chickens under lowest dropping moisture | 150-bp |
| SRP140601 | - | evaluate the effect of olive oil wastewaters as supplementation for chicken feeding | 9 chickens fed of olive oil wastewaters vs 10 control | 150-bp |
| ERP121879 | - | Exploring mRNA-seq of the jejunum mucosa in LSL and LB laying hens following the variations of dietary calcium and phosphorus levels in Lohmann LSL-Classic | 10 chickens fed with low Ca and P vs 10 control | 30-bp /101-bp |
| ERP121879 | - | Exploring mRNA-seq of the jejunum mucosa in LSL and LB laying hens following the variations of dietary calcium and phosphorus levels in Lohmann Brown-Classic | 10 chickens fed with low Ca and P vs 10 control | 30-bp /101-bp |
| **Kidney** |  |  |  |  |
| SRP097223 | (Tang et al., 2017) | Comparison of chickens raised at high and low altitudes | 3 chickens raised at high altitude vs 3 chickens raised at low altitude | 125-bp |
| SRP092600 | (Park et al., 2017) | Exploring of hypertension induced by a high calcium intake in Chicken broiler kidney under varied calcium intake | 4 chickens fed by calcium intake (1%) vs 3 chickens fed by calcium intake (1.2%) | 101-bp |
| SRP338989 | - | Exploring the effect of The injection with three IBV strains and PBS in kidney tissue of the chicken | 3 chickens with 20AD59 strain injection after 14 days vs 3 chickens with K40/06 strain injection after 14 days | 101-bp |
| SRP338989 | - | Exploring the effect of The injection with three IBV strains and PBS in kidney tissue of the chicken | 3 chickens with Kr/Q43/06 strain injection after 14 days vs 3 chickens with PBS strain injection after 14 days | 101-bp |
| SRP338989 | - | Exploring the effect of The injection with three IBV strains and PBS in kidney tissue of the chicken | 3 chickens with 20AD59 strain injection after 21 days vs 3 chickens with K40/06 strain injection after 21 days | 101-bp |
| SRP338989 | - | Exploring the effect of The injection with three IBV strains and PBS in kidney tissue of the chicken | 3 chickens with Kr/Q43/06 strain injection after 21 days vs 3 chickens with PBS strain injection after 21 days | 101-bp |
| **Liver** |  |  |  |  |
| SRP143406 | (Wei et al., 2019) | Effect of feeding regimes on egg production | 3 chickens fed ad libitum vs 3 chickens fed restricted | 125-bp |
| SRP097223 | (Tang et al., 2017) | Comparison of chickens raised at high and low altitudes | 3 chickens raised at high altitude vs 3 chickens raised at low altitude | 125-bp |
| SRP321387 | - | Exploring lipid metabolism between female chickens and male chickens | 3 male vs 3 female | 150-bp |
| SRP294224 | - | verify the function of the chicken SLCO1B3 gene on the liver metabolism | 3 chickens with blue eggshell vs 3 chickens with brown eggshel | 28-bp /101-bp |
| SRP161836 | (Zhang et al., 2020) | Exploring differential gene expression in chickens under chronic heat stress | 3 chickens under chronic heat stress vs 3 control | 150-bp |
| SRP133195 | (Ren et al., 2021) | Whole Genome Cartography of Estrogen Responsive Genes and Estrogen Receptor a Binding Sites in Chicken | 3 chickens none treated vs 3 chickens estrogen expose | 150-bp |
| SRP111815 | (Wu et al., 2018) | Identification mRNA expression profiles related to postnatal liver maturation of breeder roosters | 3 one-day-old chickens vs 3 chickens at 40weeks | 150-bp |
| SRP104528 | - | effect of dietary methionine restriction | 5 chickens fed Control diet vs 5 chickens fed L-Methionine-deficient diet | 75-bp |
| SRP233052 | (Wang et al., 2019) | Exploring evolution of cis- and trans-regulatory divergence in the Cornish chicken genome | 3 male vs 3 female | 100-bp |
| SRP233052 | (Wang et al., 2019) | Exploring evolution of cis- and trans-regulatory divergence in the White Leghorn chicken genome | 3 male vs 3 female | 100-bp |
| SRP081121 | (Li et al., 2019b) | Examination chicken under different altitudes | 6 under 3300 m vs 6 under 670 m | 125-bp |
| SRP102082 | (Zhuo et al., 2017) | Identification of Frequent of the Specific allele Expression | 3 chicken Leghorn vs 3 chicken Fayoumi | 75-bp |
| **Lung** |  |  |  |  |
| SRP097223 | (Tang et al., 2017) | Comparison of chickens raised at high and low altitudes | 3 chickens raised at high altitude vs 3 chickens raised at low altitude | 100-bp |
| SRP265640 | - | Comparison of expression profiles of SPF chicken-lung infected with duck-origin H7N9 subtype avian influenza virus with control group | 3 chickens infected by duck-origin H7N9 AIV_lung vs 3 control | 150-bp |
| SRP233531 | (Tang et al., 2020) | Transcriptome study on lung tissue of Tibetan chicken in different age | 3 chickens at 4.5 year vs 3 chickens at 5 weeks | 150-bp |
| SRP238721 | (Li et al., 2020b) | Comparative transcriptomic analysis of chicken lung infected with two different host-derived Pasteurella multocida | 3 chicken challenged with PmQ vs 3 control | 151-bp |
| SRP081121 | (Li et al., 2019b) | Examination of 4 tissues of 4 chicken types raised under different altitudes | 6 chickens raised in 3300 m vs 6 chickens raised in 670 m | 125-bp |
| **Muscle** |  |  |  |  |
| SRP217060 | (Srikanth et al., 2019) | Heat stress effect on chicken performance | 4 chickens under heat stress (5h) vs 4 control | 101-bp |
| SRP217060 | (Srikanth et al., 2019) | Heat stress effect on chicken performance | 4 chickens under heat stress (72h) vs 4 control | 101-bp |
| SRP217060 | (Srikanth et al., 2019) | Heat stress effect on chicken performance | 4 chickens under heat stress (5h) vs 4 control | 101-bp |
| SRP217060 | (Srikanth et al., 2019) | Heat stress effect on chicken performance | 4 chickens under heat stress (72h) vs 4 control | 101-bp |
| SRP159467 | (Park et al., 2018) | Comparison of chickens raised at high and low altitudes | 9 chickens raised at high altitude vs 9 chickens raised at low altitude | 75-bp |
| SRP321387 | (Li et al., 2021) | Exploring lipid metabolism between female chickens and male chickens | 3 male vs 3 female | 150-bp |
| SRP255211 | (Zhou et al., 2015) | Comparison of chickens with low and high feed efficiency | 7 chickens fed low feed efficiency vs 7 chickens fed high feed efficiency | 75-bp |
| SRP104528 | (Zhuo et al., 2017) | effect of dietary methionine restriction | 5 chickens fed L-Methionine-deficient diet vs 5 Control diet | 75-bp |
| SRP327337 | (Kubota et al., 2021) | Exploring Korat Chicken Breast Muscle with Increased Carnosine Content Produced through Dietary Supplementation with ß-Alanine | 5 chickens fed supplemented with ß-alanine vs 5 control | 150-bp |
| SRP102320 | (Li et al., 2019a) | Exploring novel candidate genes associated with meat quality at different age stages in hens | 3 chicken at 55 weekly vs 3 chicken at20 weeks old | 124-bp/150-bp |
| SRP327185 | - | Effect of dietary vitamin E on intramuscular fat deposition | 3 chickens fed a diet supplemented with 0 vitamin E vs 3 chickens fed diet supplemented with100 IU/kg vitamin E | 150-bp |
| SRP313854 | - | Chest muscle of chicken transcriptome | - | 150-bp |
| **Ovary** |  |  |  |  |
| SRP273343 | (Zhang et al., 2021) | Lingyun female chicken ovarian transcriptome sequencing | 3 chicken with high egg-laying rates vs 3 chicken with low egg-laying rates | 150-bp |
| SRP143406 | (Wei et al., 2019) | Identification of key genes and molecular mechanisms associated with low egg production of broiler breeder hens in ad libitum | 3 chickens raised in ad libitum vs 3 chickens raised in restricted | 125-bp |
| SRP256253 | (Luo et al., 2020) | Exploring the effect of FOXL2 Affects Cell Proliferation, Cycle, and DNA Replication in Chicken pre-ovulatory Follicles Cells | 3 FOXL2 kd vs 3 control | 91-bp/150-bp |
| SRP256253 | (Luo et al., 2020) | Exploring the effect of FOXL2 Affects Cell Proliferation, Cycle, and DNA Replication in Chicken pre-hierarchical Follicles Cells | 3 FOXL2 kd vs 3 control | 90-bp/150-bp |
| **Skin** |  |  |  |  |
| SRP343295 | - | Comparison of chicken groups with high abdominal fat and chicken groups with low abdominal fat | 3 high abdominal fat groups vs 3 low abdominal fat groups | 150-bp |
| SRP142597 | - | Selective breeding for Silkie chicken skin color based on the Lab color system | 3 chickens with Black Skin vs 3 chickens with Gray Skin | 90-bp/150-bp |
| SRP126033 | (Li et al., 2020a) | Exploring the breeding of the Xichuan black-bone chickens and identifying genes responsible for its unique phenotype | 3 Xichuan Black-bone chickens from yellow skin vs 3 Xichuan Black-bone chickens from black skin | 91-bp/150-bp |
| SRP112878 | (Luo et al., 2018) | Genome-wide association study and transcriptome analysis of the white/red earlobe color in Qingyuan Partridge chicken | 3 chicken with red earlobe skin vs 3 chicken white earlobe skin | 150-bp |
| **Spleen** |  |  |  |  |
| SRP097223 | (Tang et al., 2017) | Comparison of chickens with high-altitude adaptation with not adaptation with high-altitude | 3 chickens raised in high-altitude vs 3chickens raised in low-altitude | 125-bp |
| SRP225741 | (Guo et al., 2020) | Effects of stress on immune function in a chicken stress model | 3 control group vs 3 stress model group | 150-bp |
| SRP174144 | (Li et al., 2019c) | Comparison of uninfected chickens and chickens having Salmonella infection | 3 infected vs 3 control | 150-bp |
| SRP280208 | - | Comparison of highest and lowest dropping moisture in chickens | 4 chickens under highest dropping moisture vs 3 chickens under lowest dropping moisture | 150-bp |
| SRP158365 | (Qiu et al., 2018) | Comparison of infected chicken with avian leukosis virus and health chicken | 3 infected vs 3 control | 88-bp/125-bp |
| SRP174144 | (Li et al., 2019c) | Comparison of uninfected chickens and chickens having Salmonella infection | 3 infected vs 3 control | 150-bp |
| SRP254842 | - | Comparison of infected chicken with Newcastle disease virus and heat stress with uninfected chicken | 4 infected vs 4 control | 150-bp |
| SRP254842 | - | Comparison of infected chicken with Newcastle disease virus and heat stress with uninfected chicken | 4 infected vs 4 control | 150-bp |
| SRP223412 | - | Comparison of MDV-Infected chickens with Uninfected chickens | 7 infected vs 7 control | 100-bp |
| SRP173965 | (You et al., 2019) | Comparison of Marek's disease infected chickens with Uninfected chickens | 5 infected vs 5 control | 150-bp |
| SRP174144 | (Li et al., 2019c) | Comparison of uninfected chickens and chickens having Salmonella infection | 3 infected vs 3 control | 150-bp |
| SRP174144 | (Li et al., 2019c) | Comparison of uninfected chickens and chickens having Salmonella infection | 3 infected vs 3 control | 150-bp |
| **Trachea** |  |  |  |  |
| SRP338989 | - | Exploring the effect of The injection with three IBV strains and PBS in Trachea tissue of the chicken | 3 chicken with Kr/Q43/06 strain injection after 3 days vs 3 chicken with PBS strain injection after 3 days | 101-bp |
| SRP247563 | (Kulappu Arachchige et al., 2021) | Transcriptome analysis of long-term protective immunity induced by vaccination with Mycoplasma gallisepticum strain ts-304 | 3 vaccinated chicken vs 3 control | 32-bp /151-bp |
| SRP226600 | (Kulappu Arachchige et al., 2020) | The differential response of the chicken trachea to chronic infection with virulent Mycoplasma gallisepticum strain Ap3AS and Vaxsafe MG (strain ts-304) | 3 Vaccinated and challenged chicken vs 3 Challenged only chicken | 69-bp /151-bp |
| SRP126851 | (Ren et al., 2018) | Exploring differentially expressed profiles of mRNAs during Cryptosporidium baileyi infection | 3 chicken with Cryptosporidium baileyi infection vs 3 control chicken | 150-bp |

**Reference**

Boo, S. Y., Tan, S. W., Alitheen, N. B., Ho, C. L., Omar, A. R., and Yeap, S. K. (2020). Identification of reference genes in chicken intraepithelial lymphocyte natural killer cells infected with very-virulent infectious bursal disease virus. *Scientific reports* 10, 1–9.

Chanthavixay, G., Kern, C., Wang, Y., Saelao, P., Lamont, S. J., Gallardo, R. A., et al. (2020). Integrated transcriptome and histone modification analysis reveals NDV infection under heat stress affects bursa development and proliferation in susceptible chicken line. *Frontiers in genetics*, 1176.

Chen, F., Zhang, H., Du, E., Fan, Q., Zhao, N., Jin, F., et al. (2021). Supplemental magnolol or honokiol attenuates adverse effects in broilers infected with Salmonella pullorum by modulating mucosal gene expression and the gut microbiota. *Journal of Animal Science and Biotechnology* 12, 1–15.

Dai, M., Zhao, L., Li, Z., Li, X., You, B., Zhu, S., et al. (2021). The Transcriptional Differences of Avian CD4+ CD8+ Double-Positive T Cells and CD8+ T Cells From Peripheral Blood of ALV-J Infected Chickens Revealed by Smart-Seq2. *Frontiers in cellular and infection microbiology* 11.

Geng, W., Long, S. L., Chang, Y.-J., Saxton, A. M., Joyce, S. A., and Lin, J. (2020). Evaluation of bile salt hydrolase inhibitor efficacy for modulating host bile profile and physiology using a chicken model system. *Scientific reports* 10, 1–20.

Guo, Y., Jiang, R., Su, A., Tian, H., Zhang, Y., Li, W., et al. (2020). Identification of genes related to effects of stress on immune function in the spleen in a chicken stress model using transcriptome analysis. *Molecular Immunology* 124, 180–189.

Hou, L., Chen, X., Wang, J., Li, J., and Yang, H. (2021). A tandem mass tag-based quantitative proteomic analysis of fowl adenovirus serotype 4-infected LMH cells. *Veterinary Microbiology* 255, 109026.

Jin, W., Zhao, Y., Zhai, B., Li, Y., Fan, S., Yuan, P., et al. (2021). Characteristics and expression profiles of circRNAs during abdominal adipose tissue development in Chinese Gushi chickens. *PloS one* 16, e0249288.

Kubota, S., Promkhun, K., Sinpru, P., Suwanvichanee, C., Molee, W., and Molee, A. (2021). RNA Profiles of the Korat Chicken Breast Muscle with Increased Carnosine Content Produced through Dietary Supplementation with β-Alanine or L-Histidine. *Animals* 11, 2596.

Kulappu Arachchige, S. N., Young, N. D., Kanci Condello, A., Omotainse, O. S., Noormohammadi, A. H., Wawegama, N. K., et al. (2021). Transcriptomic analysis of long-term protective immunity induced by vaccination with Mycoplasma gallisepticum strain ts-304. *Frontiers in immunology* 11, 3743.

Kulappu Arachchige, S. N., Young, N. D., Shil, P. K., Legione, A. R., Kanci Condello, A., Browning, G. F., et al. (2020). Differential response of the chicken trachea to chronic infection with virulent Mycoplasma gallisepticum strain ap3as and vaxsafe mg (strain ts-304): a transcriptional profile. *Infection and immunity* 88, e00053-20.

Lee, M., Park, H., Heo, J. M., Choi, H. J., and Seo, S. (2021). Multi-tissue transcriptomic analysis reveals that L-methionine supplementation maintains the physiological homeostasis of broiler chickens than D-methionine under acute heat stress. *PLoS ONE* 16, 1–17. doi:10.1371/journal.pone.0246063.

Li, D., Li, F., Jiang, K., Zhang, M., Han, R., Jiang, R., et al. (2019a). Integrative analysis of long noncoding RNA and mRNA reveals candidate lncRNAs responsible for meat quality at different physiological stages in Gushi chicken. *PLoS One* 14, e0215006.

Li, D., Li, Y., Li, M., Che, T., Tian, S., Chen, B., et al. (2019b). Population genomics identifies patterns of genetic diversity and selection in chicken. *BMC genomics* 20, 1–12.

Li, D., Sun, G., Zhang, M., Cao, Y., Zhang, C., Fu, Y., et al. (2020a). Breeding history and candidate genes responsible for black skin of Xichuan black-bone chicken. *BMC genomics* 21, 1–15.

Li, J., Yang, C., Ren, P., Lin, Z., Zhang, D., Jiang, X., et al. (2021). Transcriptomics analysis of Daheng broilers reveals that PLIN2 regulates chicken preadipocyte proliferation, differentiation and apoptosis. *Molecular Biology Reports* 48, 7985–7997.

Li, P., He, F., Wu, C., Zhao, G., Hardwidge, P. R., Li, N., et al. (2020b). Transcriptomic analysis of chicken lungs infected with avian and bovine Pasteurella multocida serotype A. *Frontiers in Veterinary Science*, 452.

Li, X., Nie, C., Liu, Y., Chen, Y., Lv, X., Wang, L., et al. (2019c). A genome-wide association study explores the genetic determinism of host resistance to Salmonella pullorum infection in chickens. *Genetics Selection Evolution* 51, 1–12.

Luo, W., Gu, L., Li, J., and Gong, Y. (2020). Transcriptome sequencing revealed that knocking down FOXL2 affected cell proliferation, the cell cycle, and DNA replication in chicken pre-ovulatory follicle cells. *PloS one* 15, e0234795.

Luo, W., Xu, J., Li, Z., Xu, H., Lin, S., Wang, J., et al. (2018). Genome-wide association study and transcriptome analysis provide new insights into the white/red earlobe color formation in chicken. *Cellular Physiology and Biochemistry* 46, 1768–1778.

Ou, C., Wang, Q., Zhang, Y., Kong, W., Zhang, S., Yu, Y., et al. (2017). Transcription profiles of the responses of chicken bursae of Fabricius to IBDV in different timing phases. *Virology journal* 14, 1–10.

Park, J., Kim, J., and Park, W. (2018). Environmental stress to Ethiopia indigenous chicken breeds 563 induce immune signaling response among transcriptomic changes in heart, breast 564 muscle and spleen tissues. *Public on Sep* 13.

Park, W., Rengaraj, D., Kil, D. Y., Kim, H., Lee, H. K., and Song, K. D. (2017). RNA-seq analysis of the kidneys of broiler chickens fed diets containing different concentrations of calcium. *Scientific Reports* 7, 1–11. doi:10.1038/s41598-017-11379-7.

Qiu, L., Chang, G., Bi, Y., Liu, X., and Chen, G. (2018). Circular RNA and mRNA profiling reveal competing endogenous RNA networks during avian leukosis virus, subgroup J-induced tumorigenesis in chickens. *PLoS ONE* 13, 1–15. doi:10.1371/journal.pone.0204931.

Ren, G.-J., Fan, X.-C., Liu, T.-L., Wang, S.-S., and Zhao, G.-H. (2018). Genome-wide analysis of differentially expressed profiles of mRNAs, lncRNAs and circRNAs during Cryptosporidium baileyi infection. *BMC genomics* 19, 1–15.

Ren, J., Tian, W., Jiang, K., Wang, Z., Wang, D., Li, Z., et al. (2021). Global investigation of estrogen-responsive genes regulating lipid metabolism in the liver of laying hens. *BMC genomics* 22, 1–14.

Roux, P.-F., Frésard, L., Boutin, M., Leroux, S., Klopp, C., Djari, A., et al. (2016). The extent of mRNA editing is limited in chicken liver and adipose, but impacted by tissular context, genotype, age, and feeding as exemplified with a conserved edited site in COG3. *G3: Genes, Genomes, Genetics* 6, 321–335.

Srikanth, K., Kumar, H., Park, W., Byun, M., Lim, D., Kemp, S., et al. (2019). Cardiac and skeletal muscle transcriptome response to heat stress in Kenyan chicken ecotypes adapted to low and high altitudes reveal differences in thermal tolerance and stress response. *Frontiers in Genetics*, 993.

Tang, Q., Gu, Y., Zhou, X., Jin, L., Guan, J., Liu, R., et al. (2017). Comparative transcriptomics of 5 high-altitude vertebrates and their low-altitude relatives. *GigaScience* 6, 1–9. doi:10.1093/gigascience/gix105.

Tang, R., Wang, J., Zhou, M., Lan, Y., Jiang, L., Price, M., et al. (2020). Comprehensive analysis of lncRNA and mRNA expression changes in Tibetan chicken lung tissue between three developmental stages. *Animal Genetics* 51, 731–740.

Wang, Q., Jia, Y., Wang, Y., Jiang, Z., Zhou, X., Zhang, Z., et al. (2019). Evolution of cis-and trans-regulatory divergence in the chicken genome between two contrasting breeds analyzed using three tissue types at one-day-old. *BMC genomics* 20, 1–10.

Wei, Z., Li, P., Huang, S., Lkhagvagarav, P., Zhu, M., Liang, C., et al. (2019). Identification of key genes and molecular mechanisms associated with low egg production of broiler breeder hens in ad libitum. *BMC genomics* 20, 1–9.

Wu, S., Liu, Y., Guo, W., Cheng, X., Ren, X., Chen, S., et al. (2018). Identification and characterization of long noncoding RNAs and mRNAs expression profiles related to postnatal liver maturation of breeder roosters using Ribo-zero RNA sequencing. *BMC genomics* 19, 1–13.

Xiao, C., Deng, J., Zeng, L., Sun, T., Yang, Z., and Yang, X. (2021). Transcriptome analysis identifies candidate genes and signaling pathways associated with feed efficiency in Xiayan chicken. *Frontiers in genetics* 12, 368.

Yi, G., Yuan, J., Bi, H., Yan, W., Yang, N., and Qu, L. (2015). In-depth duodenal transcriptome survey in chickens with divergent feed efficiency using RNA-Seq. *PloS one* 10, e0136765.

You, Z., Zhang, Q., Liu, C., Song, J., Yang, N., and Lian, L. (2019). Integrated analysis of lncRNA and mRNA repertoires in Marek’s disease infected spleens identifies genes relevant to resistance. *Bmc Genomics* 20, 1–15.

Yu, C., Qiu, M., Zhang, Z., Song, X., Du, H., Peng, H., et al. (2021). Transcriptome sequencing reveals genes involved in cadmium-triggered oxidative stress in the chicken heart. *Poultry science* 100, 100932.

Zhang, Q., Luo, Y. K., Zhang, B. H., Chan, Y. Z., Huang, L. L., Wang, Y., et al. (2020). RNA-Seq Study of Hepatic Response of Yellow-Feather Chickens to Acute Heat Stress. *Annals of Animal Science* 20, 55–69. doi:10.2478/aoas-2019-0060.

Zhang, Q., Wang, P., Cong, G., Liu, M., Shi, S., Shao, D., et al. (2021). Comparative transcriptomic analysis of ovaries from high and low egg‐laying Lingyun black‐bone chickens. *Veterinary Medicine and Science* 7, 1867–1880.

Zhang, Q., Zhang, B., and Luo, Y. (2019). Cardiac transcriptome study of the effect of heat stress in yellow-feather broilers. *Italian Journal of Animal Science*.

Zhang, Y., Zhou, Y., Sun, G., Li, K., Li, Z., Su, A., et al. (2018). Transcriptome profile in bursa of Fabricius reveals potential mode for stress-influenced immune function in chicken stress model. *BMC genomics* 19, 1–12.

Zhou, N., Lee, W. R., and Abasht, B. (2015). Messenger RNA sequencing and pathway analysis provide novel insights into the biological basis of chickens’ feed efficiency. *BMC genomics* 16, 1–20.

Zhuo, Z., Lamont, S. J., and Abasht, B. (2017). RNA-Seq analyses identify frequent allele specific expression and no evidence of genomic imprinting in specific embryonic tissues of chicken. *Scientific reports* 7, 1–10.

Zou, X. Y., Zhang, M., Tu, W. J., Zhang, Q., Jin, M. L., Fang, R. D., et al. (2022). Bacillus subtilis inhibits intestinal inflammation and oxidative stress by regulating gut flora and related metabolites in laying hens. *Animal* 16, 100474.
