# Supplementary material for: Investigation of chicken housekeeping genes using next-generation sequencing data
Source: Front Genet. 2022 Sep 13;13:827538. doi: 10.3389/fgene.2022.827538 (PMC9514876; doi:10.3389/fgene.2022.827538)
Supplement: Supplementary file 4 [file Table2.DOCX]

| **Table 2. Top ten most suitable reference genes for chicken tissues based on average CVs across at least three experiments** | | | | | | | | | | | | | | | |
| --- | --- | --- | --- | --- | --- | --- | --- | --- | --- | --- | --- | --- | --- | --- | --- |
| **Adipose** | **Blood** | **Brain** | **Bursa** | **Duodenum** | **Heart** | **Ileum** | **Jejunum** | **Kidney** | **Liver** | **Lung** | **Muscle** | **Ovary** | **Skin** | **Spleen** | **Trachea** |
| Abcb6 | Psma1 | Serbp1 | Rap2c | Amot | Mrpl33 | Dhx30 | **Atp5b** | Itgb1bp3 | Fam120a | **Atl1** | Adam17 | **Tasor2** | **Hnrnpab** | Wdr81 | **Tasor2** |
| Prrc2c | *Gapdh* | Nr1h3 | Tmem259 | **Xpo5** | **Ilf2** | Dhx38 | *Rpl6* | Uck1 | Xpo6 | Tfip11 | Slc39a3 | Pcif1 | Ddb1 | Hdac1 | **Atl1** |
| **Ubr7^*^** | *Oaz1* | Bet1l | Cnot9 | **Exosc10** | Rufy3 | Arnt | **Gnb2l1** | *Polr2b* | **Ubr7** | Mvb12a | **Cops7a** | Tomm22 | Lonp1 | Scyl3 | **Ikbkb** |
| Eif3a | Rpl39l | Arhgef9 | Wasf2 | Zyx | **Cep68** | Ckap2l | **Ilf2** | Col4a1 | Mrps25 | Slc35a1 | Xpo7 | Erlin1 | Tcf25 | Zc3h11b | **Cep68** |
| Pabpc1 | Tmed10 | **Atp5b** | **Grb2** | Usp5 | Nfyc | **Hnrnpab** | Lasp1 | *Gusb* | *Ap2m1* | Pisd | Fem1b | Poll | **Grb2** | Dnajc5 | Cfap92 |
| Tmem57 | *Rpl27a* | Nono | Ascc2 | Cnot1 | Cuedc2 | Nup188 | Mif4gd | **Atl1** | Pcbd1 | Phc1 | Ipo9 | **Ctnna1** | Mtmr3 | Nek9 | Parp9 |
| Thrap3 | Cox7b | Sumo3 | Rpl7l1 | Psmd13 | **Tasor2** | Stx10 | **Ikbkb** | Hbp1 | Rnf130 | Spout1 | **Gsr** | Kbtbd4 | Baz1b | Gzf1 | Hspd1 |
| *Rpl4* | Rpl7a | Rps3a | Man2c1 | Fhl3 | Slc7a3 | **Eif2b5** | **Atl1** | Herc2 | Adat1 | **Tor1b** | Copg2 | Brd8 | **Exosc10** | Cdk12 | Cd80 |
| **Ctnna1** | Rpsap58 | Uso1 | Tpm3 | Med1 | **Gnb2l1** | Map3k14 | Mkrn2 | Ndufb9 | Myh9 | Cog5 | Arcn1 | **Gsr** | **Cops7a** | **Xpo5** | Mettl21c |
| Kcnh4 | Eif4ebp1 | Cd99l2 | Chmp1a | **Eif2b5** | Ak2 | Nol9 | Aamp | **Tor1b** | Arpc2 | **Cep68** | Eya3 | Clpx | Yipf3 | Mpp1 | Mrpl40 |
| **^*^** Highlighted (bold) genes are in common for at least two tissues. Underlined genes are among the widely used housekeeping genes. | | | | | | | | | | | | | | | |
