# Supplementary material for: Investigation of chicken housekeeping genes using next-generation sequencing data
Source: Front Genet. 2022 Sep 13;13:827538. doi: 10.3389/fgene.2022.827538 (PMC9514876; doi:10.3389/fgene.2022.827538)
Supplement: Supplementary file 6 [file Table4.docx]

| Table 4. Most stably expressed genes of five chicken tissues that are in common between the top-100 genes of training and top-100 genes of evaluation datasets | | | | |
| --- | --- | --- | --- | --- |
| Heart | Kidney | Liver | Muscle | Spleen |
| Mob1a | Ikbkb | Rp11-529k1.3 | Srpra | Nfyc |
| **Strada** | Wnk1 | Dpagt1 | Cops7a | Hdac1 |
| Ticam1 | Aplp2 | Rbm7 | Hnrnpd | Cnp |
| Cep68 | Ilf2 | Npepps | Dhx38 | Adam17 |
| **Phc1** | **Strada** | Eif2b5 | Gtpbp1 | Spata5 |
| **Atl1** | Ugp2 | *Ap2m1* | Puf60 | Grk2 |
| Fancm | Bpnt1 | Amfr |  | Rpn1 |
| Abi2 | **Phc1** | Psmd7 |  | Hnrnpab |
| Aamp | Etfdh | Fam120a |  | Mtmr3 |
| Tmem41a | Rufy3 | Ctnna1 |  | Nup188 |
| Ak3 | Oraov1 | Xpo6 |  | Tor1b |
| Hbp1 | Slirp | Lig3 |  | Znrf2 |
| Casc4 | **Atl1** | **Ankrd16** |  | **Ankrd16** |
| Rbl2 | Hspd1 | Prpf6 |  |  |
| Pepd | Tanc1 |  |  |  |
| **Mkrn2** | *Rpl5* |  |  |  |
| Tfip11 | Cp |  |  |  |
| Pisd | Gpr18 |  |  |  |
| Spout1 | Cog5 |  |  |  |
| **Uck1** | Vwa9 |  |  |  |
| Mif4gd | **Mkrn2** |  |  |  |
| **Ankrd16** | Hvcn1 |  |  |  |
| Tasor2 | **Uck1** |  |  |  |
|  | Gsn |  |  |  |
|  | Fam104a |  |  |  |
|  | Mrps7 |  |  |  |
|  | *Gusb* |  |  |  |
|  | Rps6kb1 |  |  |  |
|  | Myo19 |  |  |  |
|  | Tubb2a |  |  |  |
|  | Stx17 |  |  |  |
|  | Mrps16 |  |  |  |
| **^*^** Highlighted (bold) genes are in common for at least two tissues. Underlined genes are among the widely used housekeeping genes. | | | | |
